# Parental High-Fat High-Sugar Diet Intake Programming Inflammatory and Oxidative Parameters of Reproductive Health in Male Offspring

**DOI:** 10.3389/fcell.2022.867127

**Published:** 2022-06-27

**Authors:** Marcela Nascimento Sertorio, Helena César, Esther Alves de Souza, Laís Vales Mennitti, Aline Boveto Santamarina, Leonardo Mendes De Souza Mesquita, Andréa Jucá, Breno Picin Casagrande, Debora Estadella, Odair Aguiar, Luciana Pellegrini Pisani

**Affiliations:** ^1^ Biosciences Department, Institute of Health and Society, Federal University of São Paulo, Santos, Brazil; ^2^ Multidisciplinary Laboratory of Food and Health, School of Applied Sciences (FCA), University of Campinas, Limeira, Brazil

**Keywords:** testis, epididimys, epigenetics, high-fat diet, fetal programming, inflammation, oxidative stress, sperm production

## Abstract

Parental nutrition can impact the health of future generations, programming the offspring for the development of diseases. The developing germ cells of the offspring could be damaged by the maternal or the paternal environment. The germ cells in development and their function could be affected by nutritional adversity and therefore, harm the health of subsequent generations. The paternal or maternal intake of high-fat diets has been shown to affect the reproductive health of male offspring, leading to imbalance in hypothalamic-pituitary-gonadal axis, testicular oxidative stress, low testosterone production, and changes in sperm count, viability, motility, and morphology. There is a need for studies that address the combined effects of diets with a high-fat and high-sugar (H) content by both progenitors on male reproduction. In this context, our study evaluated epigenetic parameters and the inflammatory response that could be associated to oxidative stress in testis and epididymis of adult offspring. 90 days-old male rats were divided according to the combination of the parental diet: CD (control paternal and maternal diet), HP (H paternal diet and control maternal diet), HM (H maternal diet and control paternal diet) and HPM (H paternal and maternal diet).We evaluated serum levels of testosterone and FSH; testicular gene expression of steroidogenic enzymes *Star* and *Hsd17b3* and epigenetic markers *Dnmt1*, *Dnmt3a*, *Dnmt3b*, and *Mecp2*; testicular and epididymal levels of TNF-α, IL-6, IL-10, and IL-1β; testicular and epididymal activity of SOD, CAT, and GST; the oxidative markers MDA and CP; the daily sperm production, sperm transit time, and sperm morphology. Testicular epigenetic parameter, inflammatory response, oxidative balance, and daily sperm production of the offspring were affected by the maternal diet; paternal diet influenced serum testosterone levels, and lower daily sperm production was exacerbated by the interaction effect of both parental intake of high-fat high-sugar diet in the testis. There was isolated maternal and paternal effect in the antioxidant enzyme activity in the cauda epididymis, and an interaction effect of both parents in protein oxidative marker. Maternal effect could also be observed in cytokine production of cauda epididymis, and no morphological effects were observed in the sperm. The potential programming effects of isolated or combined intake of a high-fat high-sugar diet by the progenitors could be observed at a molecular level in the reproductive health of male offspring in early adulthood.

## 1 Introduction

Inadequate nutrition through the intake of high-fat and high-sugar diets can set adverse issues for the health as an individual, and also for the health of next generations. According to the Developmental Origins of Health and Disease (DOHaD), adverse events in early stages of embryonic development could program the pattern of health and disease during the life of the offspring, a phenomenon known as fetal programming (Barker, 1998; Jazwiec and Sloboda, 2019). Parental inadequate nutrition has been shown to program the offspring for the development of diseases, given that the proper development and maturation of organs and systems in the early stages of life relies on healthy environments (Barker, 1998; Fleming et al., 2018; Jazwiec and Sloboda, 2019). Fetal programming can result from epigenetic modifications, that consist in heritable changes in gene expression with no alteration in DNA nucleotide sequence, such as covalent modifications in histone lysine residues, DNA methylation, and post-transcriptional changes (Reid et al., 2017; Safi-Stibler and Gabory, 2020). The parental intake of high-fat and high-sugar diets has been shown to influence the development of long-term chronic diseases such as cardiovascular disease, insulin resistance, hypertension and obesity in the offspring through epigenetics mechanisms (Fleming et al., 2018; Radford, 2018; Jazwiec and Sloboda, 2019).

The germ cells in development could also be affected by nutritional adversity, and experimental data reveals that parental intake of high-fat diets could harm the quality of reproductive parameters in both female and male offspring (Crean and Senior, 2019; Jazwiec and Sloboda, 2019; Sertorio et al., 2021). This issue becomes even more relevant due to the fact that offspring gametes of poor quality could still impair the metabolic health of subsequent generations (Radford, 2018). Parental high-fat diet intake prior to conception and/or during gestation and lactation seem to influence the reproductive health of male offspring (Sertorio et al., 2021). Adipose tissue hypertrophy leads to the production of adipokines, unbalancing the hypothalamic-pituitary-gonadal axis and impairing the production of gonadotropins (Jacobs et al., 2014; Rodríguez-González et al., 2015; Sanchez-Garrido et al., 2018); this impairment could then drive changes in the gene expression of steroidogenic acute regulatory protein (StAR) and 17β-hydroxysteroid dehydrogenase enzyme (17β-HSD), key steroidogenic enzymes of the testosterone production cascade (Sanchez-Garrido et al., 2018). The parental intake of high-fat diets could also program testicular oxidative imbalance in the offspring, disrupting the activity of the antioxidant enzymes catalase (CAT), superoxide dismutase (SOD) and glutathione, with the production of cell damage markers, such as malondialdehyde (MDA) (Rodríguez-González et al., 2015; Bautista et al., 2017), harming testosterone production and spermatogenesis (Christante et al., 2013; Reame et al., 2014). Consequently, the sperm count, viability and motility, besides sperm morphology in the epididymis could be compromised (Fullston et al., 2015; Rodríguez-González et al., 2015). Changes in molecular sperm parameters could predict lower sperm quality and therefore, poor development of a potential zygote, setting risk for more than one generation (Palmer et al., 2012; Fullston et al., 2013; Fleming et al., 2018).

Adipose tissue accumulation high-fat diet-induced can be related to altered DNA methylation profile in the germline of adult male rats, such as altered expression pattern of DNA methyltransferases (DNMTs) in the testis (Deshpande et al., 2020). It can also be related to the development of an inflammatory state in the male genital tract, with the production of the cytokines tumor necrosis factor alpha (TNF-α), interleukin 6 (IL-6), interleukin 1β (IL-1β), and interleukin 10 (IL-10) (Tremellen, 2016; Fan et al., 2018; Yi et al., 2020). The inflammatory response induced by adiposity could generate reactive oxygen species (ROS), overloading the antioxidant capacity in the testis, and reducing sperm quality (Yi et al., 2020). To our knowledge, this is the first programming study addressing epigenetic and inflammatory changes in the reproductive health of male offspring from progenitors fed on high-fat high-sugar diet, as well as the additive impact of high-fat diets intake by both progenitors on male reproduction. Our objective was to assess the parental high-fat high-sugar epigenetic programming on male reproductive health in testis and epididymis of adult rats, focusing on the link between the inflammatory response and oxidative stress over sperm production in the offspring.

## 2 Materials and Methods

### 2.1 Animals and Diet

All animals were housed in polypropylene cages under controlled lighting (light/dark cycle) and temperature (22°C) with free access to its proper diet and drinking water. Male Wistar rats (8-weeks-old) were submitted to the intake of a control (*n* = 11) or a modified diet (*n* = 11), prior to conception during 10 weeks. Female Wistar rats (10-weeks-old) also received a control or modified diet during gestation and lactation. Males and females were randomly placed to receive control diet (CD) (15.5% fat; 50% carbohydrates, Nuvilab CR1, Quimtia®) or high-fat high-sugar diet (H) [32% fat; 50% carbohydrates (25% from sugar)]. Sweetened condensed milk was the main source of sugar and lard the main source of fat in H diet. Vitamin and mineral mix were added to avoid micronutrient deficiencies, as well as choline bitartrate and L-cysteine in order to provide the quantity of essential nutrients of CD diet (César et al., 2021).

After 10 weeks of diet intake, males were mated to females. When copulation was confirmed, females were fed CD or H diet during gestation and lactation. The period of 10 weeks comprises a whole cycle of spermatogenesis for male rats, assuring this way, that all germ cells were exposed to the treatment. This period is also considered a chronic treatment for inducing increased adiposity in rats. Gestation is the most important period of growth and development, and maternal modified diet during this period has been shown to program the development of offspring. The following groups were formed: CD (control paternal and maternal diet, *n* = 6), HP (H paternal diet and control maternal diet, *n* = 5), HM (H maternal diet and control paternal diet, *n* = 5) and HPM (H paternal and maternal diet, *n* = 6). Eight male pups were used for each litter per dam, and after weaning, the male pups were fed a control diet during 90 days. 1–2 pups per litter were used in the experimental analyses. Each individual male offspring was treated as a biological replicate for statistics.

### 2.2 Euthanasia and Material Collection

Male progenitors were euthanized after mating, female progenitors after 21 days of weaning and male offspring at 90 days old. The animals were fasted for 10–12 h prior to euthanasia, and placed in anesthesia jar containing cotton soaked in isoflurane. The cotton pad was separated through a physical barrier to avoid direct contact between animal and the anesthetic. After confirmation of lack of reflex and reduced respiratory rate, animals were euthanized by decapitation under deep anesthesia. All the procedures were performed following the rules issued by the National Council for Control of Animal Experimentation (CONCEA). The use of animals for this study was approved by the Ethic Committee on Animal Use of the Federal University of São Paulo (CEUA number 9856031018/UNIFESP).

Trunk blood was collected and centrifuged at 700 x g in 4°C for 15 min. Serum was collected, frozen and stored (−80°C). Retroperitoneal (RET), mesenteric (MES), and epididymal (EPI) adipose tissue, testis, and epididymis were weighted, frozen and stored (−80°C). Sperm from the vas deferens was collected and fixed in 4% buffered formaldehyde for morphologic analysis.

### 2.3 Quantification of Serum Testosterone and FSH Levels

The quantification of serum levels of testosterone and FSH was performed through chemiluminescence assay by the clinical analysis laboratory Hermes Pardini, SP, Brazil.

### 2.4 Gene Expression of Steroidogenic Enzymes and Epigenetic Markers

Extraction of total RNA from testis samples was performed using trizol reagent (TRI-reagent, Sigma, St. Louis, MO, United States), according to the manufacturer’s instructions. To obtain the concentration of RNA/μl, the reading was performed at wavelengths of 260, 280 and 230 nm. The degree of purity was estimated by the 260/280 nm ratio which must vary between 1.8 and 2.0 for nucleic acids. Total RNA was quantified using the NanoDrop ND-1000 spectrophotometer (NanoDrop Technologies Inc., Wilmington, United States). For cDNA generation, the total RNA of each sample was subjected to reverse transcription, using the M- MLV Reverse Transcriptase Kit (PROMEGA, Madison, WI, United States). Relative levels of mRNA for *Star* and *Hsd17b3* in the testis were quantified using the PCR-RT technique using the ABI Prism 7500 Sequence Detector (Applied Biosystems, Foster City, CA, United States). Primers sequences of Beta-actin, StAR and 17β-HSD were obtained from the U.S National Library of Medicine Primer designing tool (https://www.ncbi.nlm.nih.gov/tools/primer-blast/). To assess stability of the primers in the samples, amplification efficiency curves were determined. Gene sequences of DNA methyltransferase 1 (*Dnmt1*), DNA methyltransferase 3a (*Dnmt3a*), DNA methyltransferase 3b (*Dnmt3b*) and Methyl CpG binding protein 2 (*Mecp2*) were used according to previous study from our research group (Santamarina et al., 2018). Primer sequences used in the study are displayed in Table 1. The detection method used was the SyberGreen fluorophore (Applied Biosystems, Foster City, CA, United States). Relative mRNA levels for *Hprt* (hypoxanthine phosphoribosyltransferase-1) and Beta-actin were determined as reference genes. The information was recorded using the Sequence Detector software (Applied Biosystems, Foster City, CA, United States).

**TABLE 1:** Primer sequences used in the study.

Genes	Sequences
*Beta-actin* F	CTA​AGG​CCA​ACC​GTG​AAA​AGA
*Beta-actin* R	CCA​GAG​GCA​TAC​AGG​GAC​AAC
*Hprt* F	CTC​ATG​GAC​TGA​TTA​TGG​ACA​GGA​C
*Hprt* R	GAC​GGT​CAG​CAA​GAA​CTT​ATA​GCC
*Star* F	AAG​GCT​GGA​AGA​AGG​AAA​GC
*Star* R	CAC​CTG​GCA​CCA​CCT​TAC​TT
*Hsd17b3* F	TGA​CCA​AGA​CCG​CCG​ATG​AGT​T
*Hsd17b3* R	TGG​GTG​GTG​CTG​CTG​TAG​AAG​AT
*Dnmt1* F	TCC​TAC​GCC​ATG​CCC​AGT​TTG
*Dnmt1* R	GAA​GAT​GGG​CGT​CTC​ATC​ATC​G
*Dnmt 3a* F	GCC​CAT​TCG​ATC​TGG​TGA​TTG
*Dnmt 3a* R	TCG​TAA​AGT​CCC​TTG​CGG​GC
*Dnmt 3b* F	TGT​GCA​GAG​TCC​ATT​GCT​GTA​GGA
*Dnmt 3b* R	GCT​TCC​GCC​AAT​CAC​CAA​GTC​AAA
*Mecp2* F	CAG​CTC​CAA​CAG​GAT​TCC​ATG​GT
*Mecp2* R	TGA​TGT​CTC​TGC​TTT​GCC​TGC​CT

F, forward; R, reverse.

### 2.5 Extraction of Proteins in the Testis and Epididymis

Testis and epididymis samples (200 mg) were homogenized in 0.6 ml of extraction buffer (100 mM EDTA, 100 mM Tris, 10 mM sodium pyrophosphate, 100 mM sodium fluoride, 10 mM sodium orthovanadate, 2 mM phenylmethylsulphonyl fluoride, and 0.1 mg/ml aprotinin. To the homogenized samples Triton X-100 10% was added and then centrifuged after 30 min at 22.600 × g for 40 min at 4°C in order to collect the supernatant. (César et al., 2021). The supernatant was used to quantify the cytokine levels and total protein content, according to the Bradford method (Bradford, 1976). Samples were kept at −80°C.

### 2.6 Levels of TNF-α, IL-6, IL-1β, and IL-10 in the Testis and Epididymis

The levels of the cytokines TNF-α, IL-6, IL-1β, and IL-10 in the testis and epididymis were determined using the ELISA method using R&D Systems kits, following the manufacturer’s instructions. For detection limit, standard curves were calculated for each cytokine. The standard curve for TNF-α (DY510), IL-10 (DY522), and IL-1β (DY501) ranged from 62.5 to 4.000 pg/ml, and IL-6 (DY506) ranged from 125 to 8.000 pg/ml.

### 2.7 Spermatid Number, Daily Sperm Production, Sperm Number and Transit Time

Spermatids resistant to homogenization in the testis and sperm resistant to homogenization in the caput/corpus and cauda epididymis were counted in Newbauer chambers (four fields per animal), after homogenization of the organ in a solution of 0.9% NaCl and 0.05% Triton X-100 (Robb et al., 1978).

To determine the daily sperm production, the number of spermatids per testis was divided by 6.1, which corresponds to the number of days in which mature spermatids are present in the seminiferous epithelium. For sperm transit time in the caput/corpus and cauda epididymis in days, the number of sperm in each portion was divided by the daily sperm production (Robb et al., 1978).

### 2.8 Sperm Morphology

Sperm morphology was performed using sperm from spermatic fluid of the vas deferens and fixed in 4% buffered formaldehyde. The solution was applied to histological slides for analysis of two hundred sperm per sample under a Nikon Eclipse E100 microscope (Tokyo, Japan) in a 40x magnification lens. Sperm were classified as normal or with head or tail defects (Filler, 1993).

### 2.9 Antioxidant Enzyme Activity and Oxidative Stress Markers

Testis and epididymis samples (150 mg) were homogenized in phosphate buffer 0.2 M pH 7.4 (1 ml) and centrifuged at 10.000 × g at 4°C for 10 min. The supernatant was used for the analysis of CAT, SOD and glutathione S-transferase (GST) activities, MDA oxidative marker and total protein content. For the carbonyl protein (CP) oxidative marker, the pellet was used. CAT activity was estimated by measuring the decomposition of hydrogen peroxide (Góth, 1991). SOD activity was evaluated by the pyrogallol method, based on the ability of this enzyme to catalyze the reaction of superoxide and hydrogen peroxide. GST activity was estimated by the conjugation of gluthatione tiol groups to 1-chloro-2,4-dinitrobenzene (Habig et al., 1974) MDA was assessed by the formation of thiobarbituric acid reactive substances (Buege and Aust, 2007) and CP was determined by the derivatization of carbonyl groups with 2,4-dinitrophenylhydrazine, leading to the formation of dinitrophenyl hydrazones (Levine et al., 1994). Total protein content was assessed according to the Bradford method (Bradford, 1976).

### 2.10 Statistical Analysis

The normality of the data was evaluated by Shapiro–Wilk test and logarithmic transformation was used for normalization of data. The identification of outliers was performed by the Grubbs method and data were assessed by t-test for parental data and two-way ANOVA, followed by Bonferroni post-hoc test for offspring data. Results were expressed as mean and the standard error of the mean (SEM). Statistical analysis was performed with the software JASP 0.12.1.0 with a significance level of *p* < 0.05. Results were described according to the effect of the factor causing the changes (paternal or maternal diet). Groups were compared individually by the post-hoc test when the interaction between the factors was detected.

## 3 Results

### 3.1 Progenitors

#### 3.1.1 Biometric Data

Significant changes caused by the high-fat high-sugar diet intake were observed in both male and female progenitors (Supplementary Figure S1). There was an increase in female progenitor body weight at the end of gestation (*n* = 11, *p* < 0.05, Supplementary Figure S1A), but not after the lactation period (*n* = 11, *p* > 0.05, Supplementary Figure S1B) when compared to the control group. There was also an increase in the relative weight of RET compared to CD (*n* = 8, *p* < 0.05, Supplementary Figure S1C). After 10 weeks of treatment, the body weight was increased in male progenitors in comparison to CD (*n* = 11, *p* < 0.05, Supplementary Figure S1D), as well as RET adiposity (*n* = 8, *p* < 0.05, Supplementary Figure S1E).

### 3.2 Male Offspring

#### 3.2.1 Maternal Influence

**FIGURE 1:**
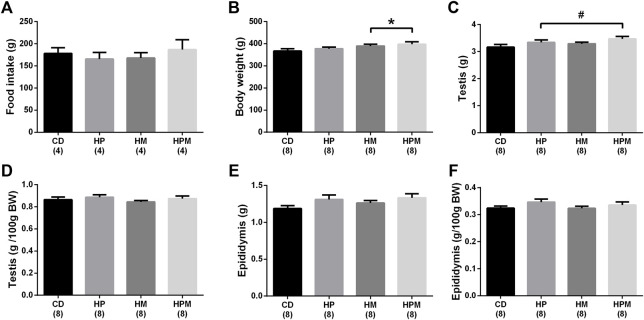
Biometric data and food intake of male rat offspring from progenitors fed on high-fat high-sugar diet. Mean ± SEM; symbols indicate significant difference caused by parental diet (*p* < 0.05): ^#^statistically significant by the effect of paternal diet; *statistically significant by the effect of maternal diet. H: high-fat and high-sugar diet; CD (control paternal and maternal diet), HP (H paternal diet and control maternal diet), HM (H maternal diet and control paternal diet) and HPM (H paternal and maternal diet); BW: body weight. **(A)**: food intake; **(B)**: body weight (g); **(C)**: testis (g); **(D)**: testis (g/100 g BW); **(E)**: epididymis (g); epididymis (g/100 g BW); (*n* = 8).

**FIGURE 2:**
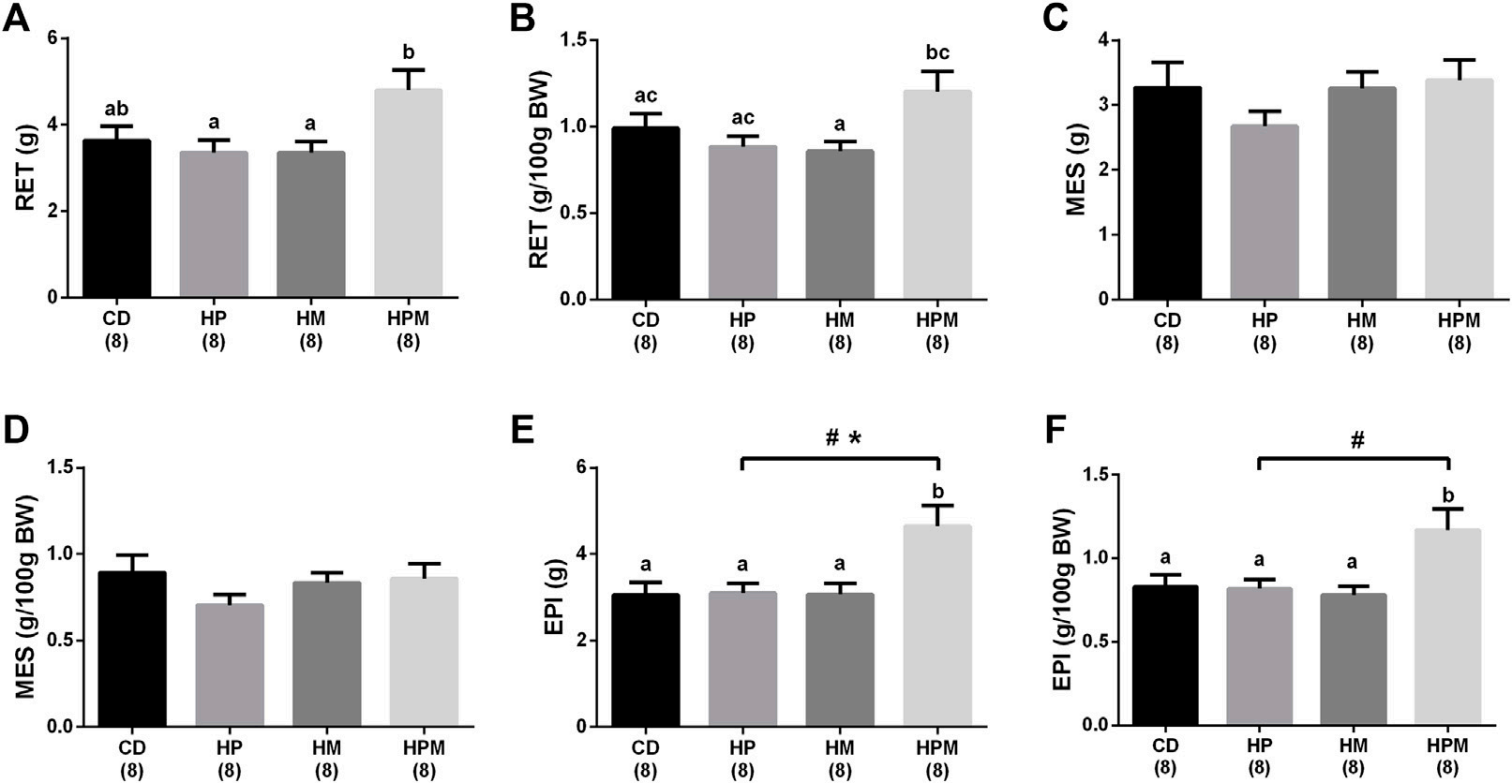
Adipose tissue data of male rat offspring from progenitors fed on high-fat high-sugar diet. Mean ± SEM; symbols indicate significant difference caused by the effect of parental diet (*p* < 0.05): ^#^statistically significant by paternal diet; *statistically significant by the effect of maternal diet. a,b,c Different letters represent significant difference between groups (*p* < 0.05). H: high-fat and high-sugar diet; CD (control paternal and maternal diet), HP (H paternal diet and control maternal diet), HM (H maternal diet and control paternal diet) and HPM (H paternal and maternal diet); BW: body weight; **(A)**: retroperitoneal adipose tissue (RET) (g); **(B)**: RET (g/100 g BW); **(C)**: mesenteric adipose tissue (MES) (g); **(D)**: MES (g/100 g BW); **(E)**: epididymal adipose tissue (EPI) (g); **(F)**: EPI (g/100 g BW); (*n* = 8).

**FIGURE 3:**
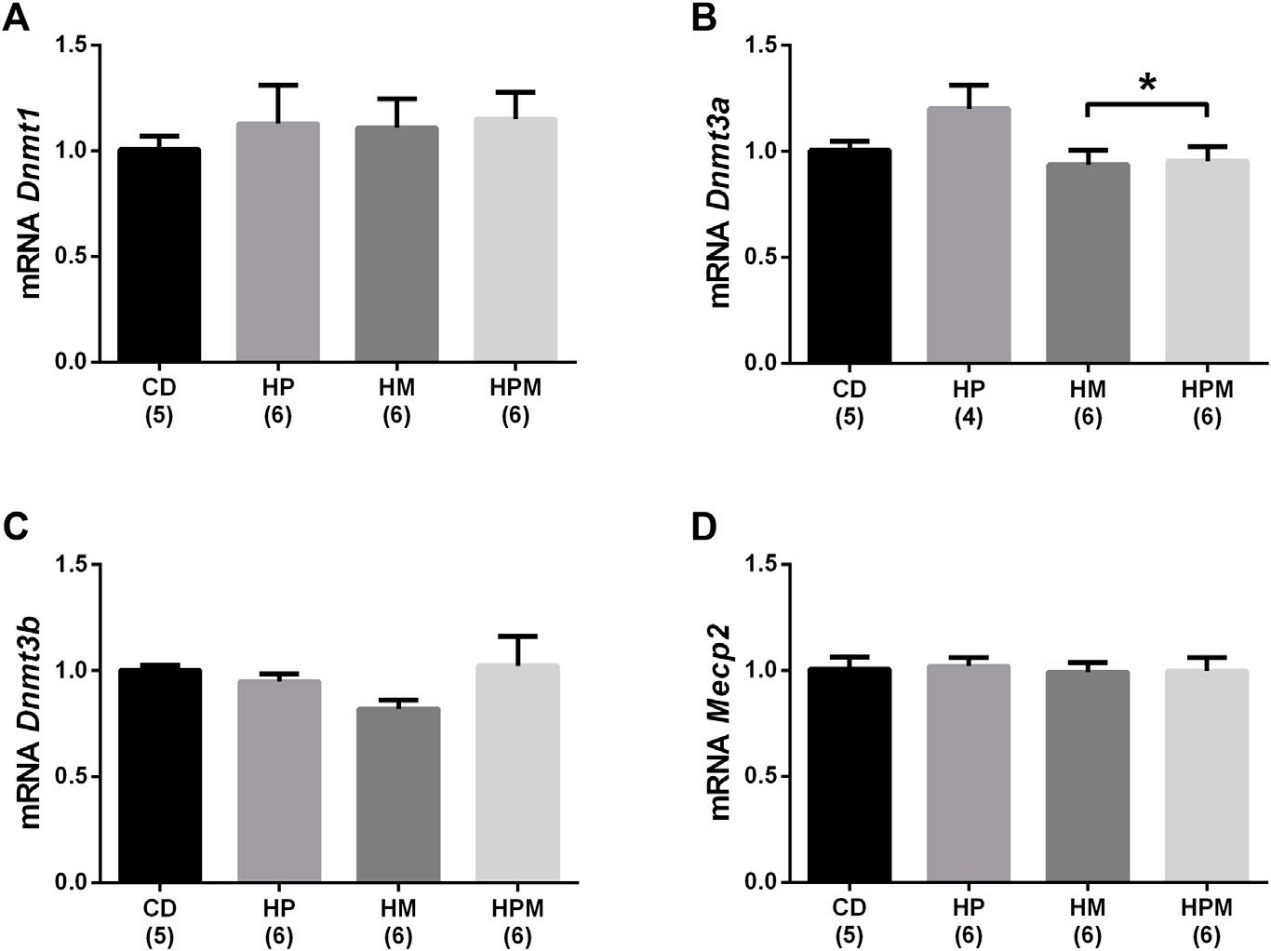
Testicular gene expression of epigenetic markers in male rat offspring from progenitors fed on high-fat high-sugar diet. Mean ± SEM; symbols indicate significant difference caused by parental diet (*p* < 0.05): *statistically significant by the effect of maternal diet. H: high-fat and high-sugar diet; CD (control paternal and maternal diet), HP (H paternal diet and control maternal diet), HM (H maternal diet and control paternal diet) and HPM (H paternal and maternal diet). **(A)**: DNA methyltransferase 1 (*Dnmt1*) (*n* = 5-6); **(B)**: DNA methyltransferase 3a (*Dnmt3a*) (*n* = 4-6); **(C)**: DNA methyltransferase 3b (*Dnmt3b*) (*n* = 5-6); **(D)**: methyl CpG binding protein 2 (*Mecp2*) (*n* = 5-6).

**FIGURE 4:**
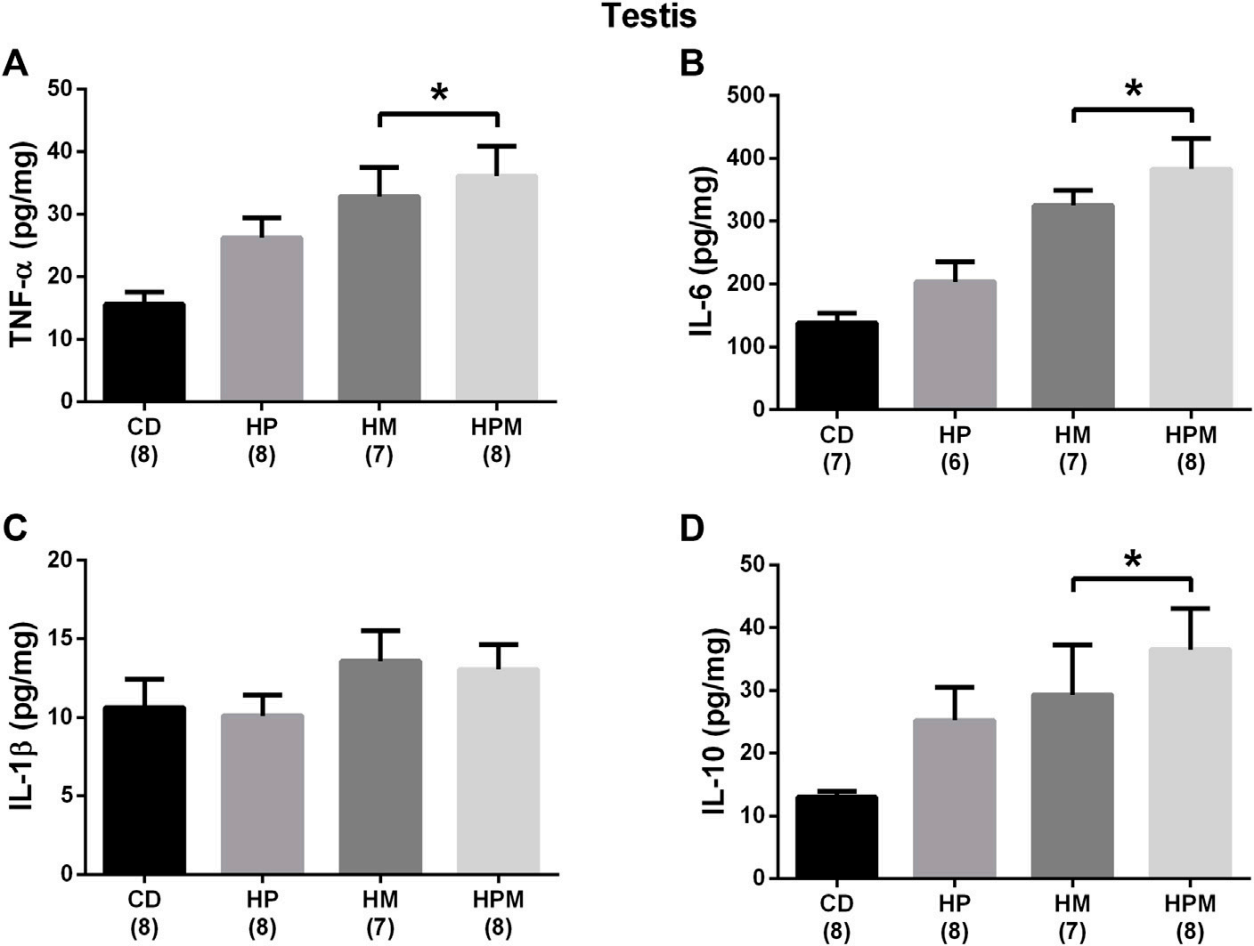
Testicular cytokine levels in male rat offspring from progenitors fed on high-fat high-sugar diet. Mean ± SEM; symbols indicate significant difference caused by parental diet (*p* < 0.05): *statistically significant by the effect of maternal diet. H: high-fat and high-sugar diet; CD (control paternal and maternal diet), HP (H paternal diet and control maternal diet), HM (H maternal diet and control paternal diet) and HPM (H paternal and maternal diet). **(A)**: tumor necrosis factor *α* (TNF-α) (pg/mg) (*n* = 7-8); **(B)**: interleukin 6 (IL-6) (pg/mg) (*n* = 6-8); **(C)**: interleukin 1β (IL-1β) (pg/mg) (*n* = 7-8); **(D)**: interleukin 10 (IL-10) (pg/mg) (*n* = 7-8).

**FIGURE 5:**
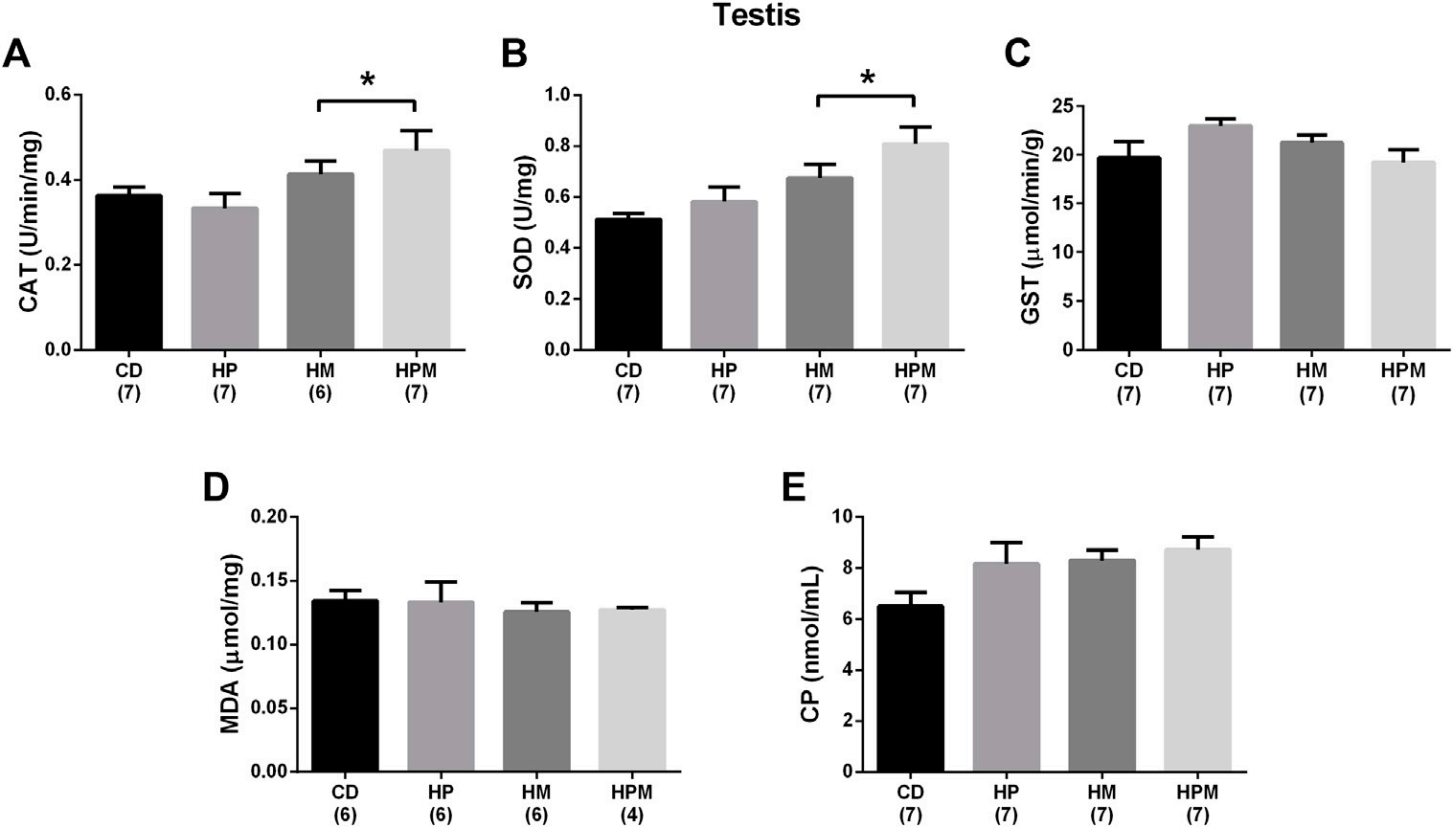
Testicular antioxidant enzyme activity and oxidative stress markers in male rat offspring from progenitors fed on high-fat high-sugar diet. Mean ± SEM; symbols indicate significant difference caused by parental diet (*p* < 0.05): *statistically significant by the effect of maternal diet. H: high-fat and high-sugar diet; CD (control paternal and maternal diet), HP (H paternal diet and control maternal diet), HM (H maternal diet and control paternal diet) and HPM (H paternal and maternal diet). **(A)** catalase (CAT) (U/min/mg) (*n* = 6-7); **(B)**: superoxide dismutase (SOD) (U/mg) (*n* = 7); **(C)**: glutathione S-transferase (GST) (μmol/min/g) (*n* = 7); **(D)**: malondialdehyde (MDA) (μmol/mg) (*n* = 4-6); **(E)**: CP (carbonyl protein) (nmol/mL) (*n* = 7).

**FIGURE 6:**
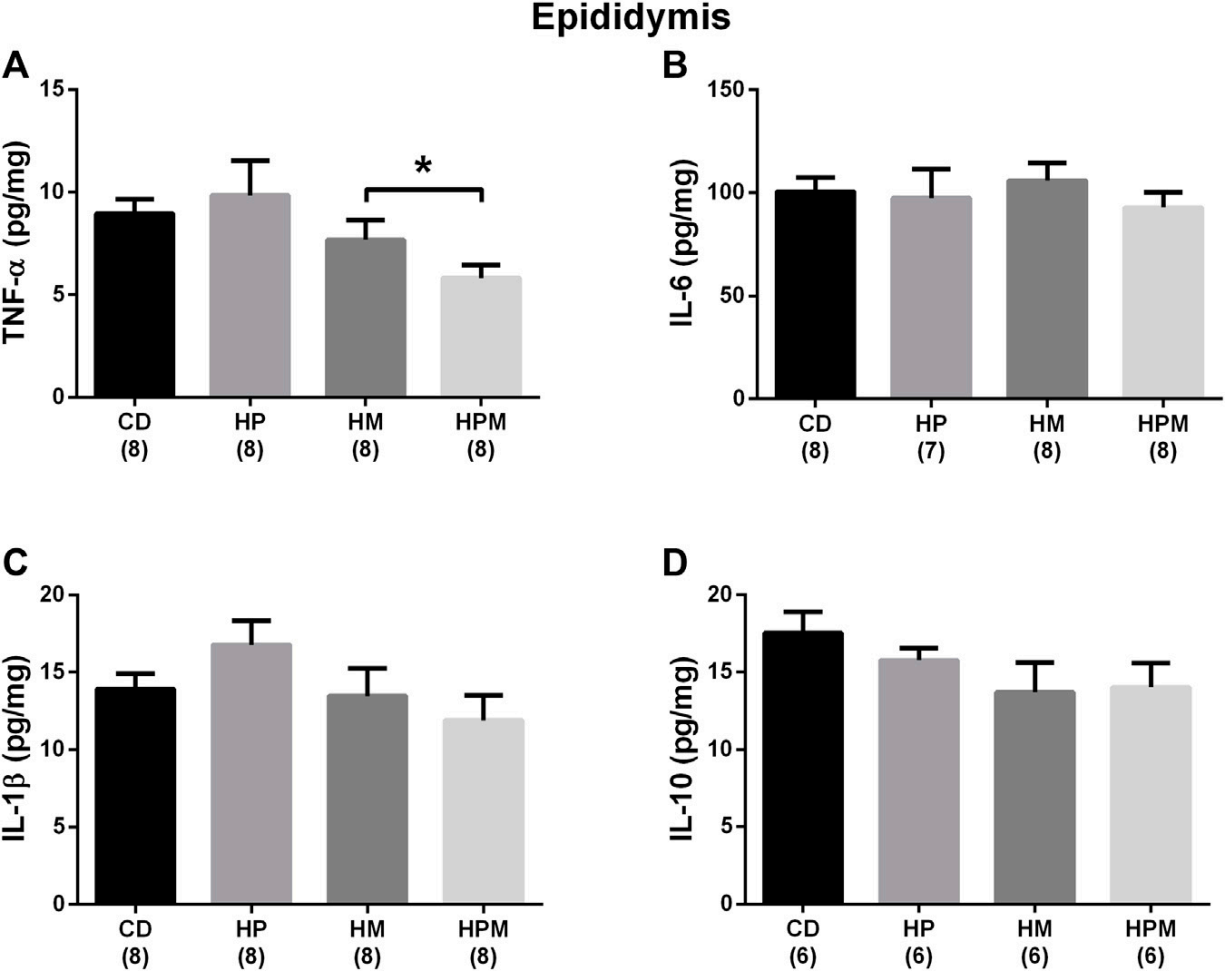
Epididymal cytokine levels in male rat offspring from progenitors fed on high-fat high-sugar diet. Mean ± SEM; symbols indicate significant difference caused by parental diet (*p* < 0.05): *statistically significant by the effect of maternal diet. H: high-fat and high-sugar diet; CD (control paternal and maternal diet), HP (H paternal diet and control maternal diet), HM (H maternal diet and control paternal diet) and HPM (H paternal and maternal diet). A: tumor necrosis factor *α* (TNF-α) (pg/mg) (*n* = 8); B: interleukin 6 (IL-6) (pg/mg) (*n* = 7-8); C: interleukin 1β (IL-1β) (pg/mg) (*n* = 8); D: interleukin 10 (IL-10) (pg/mg) (*n* = 6).

**FIGURE 7:**
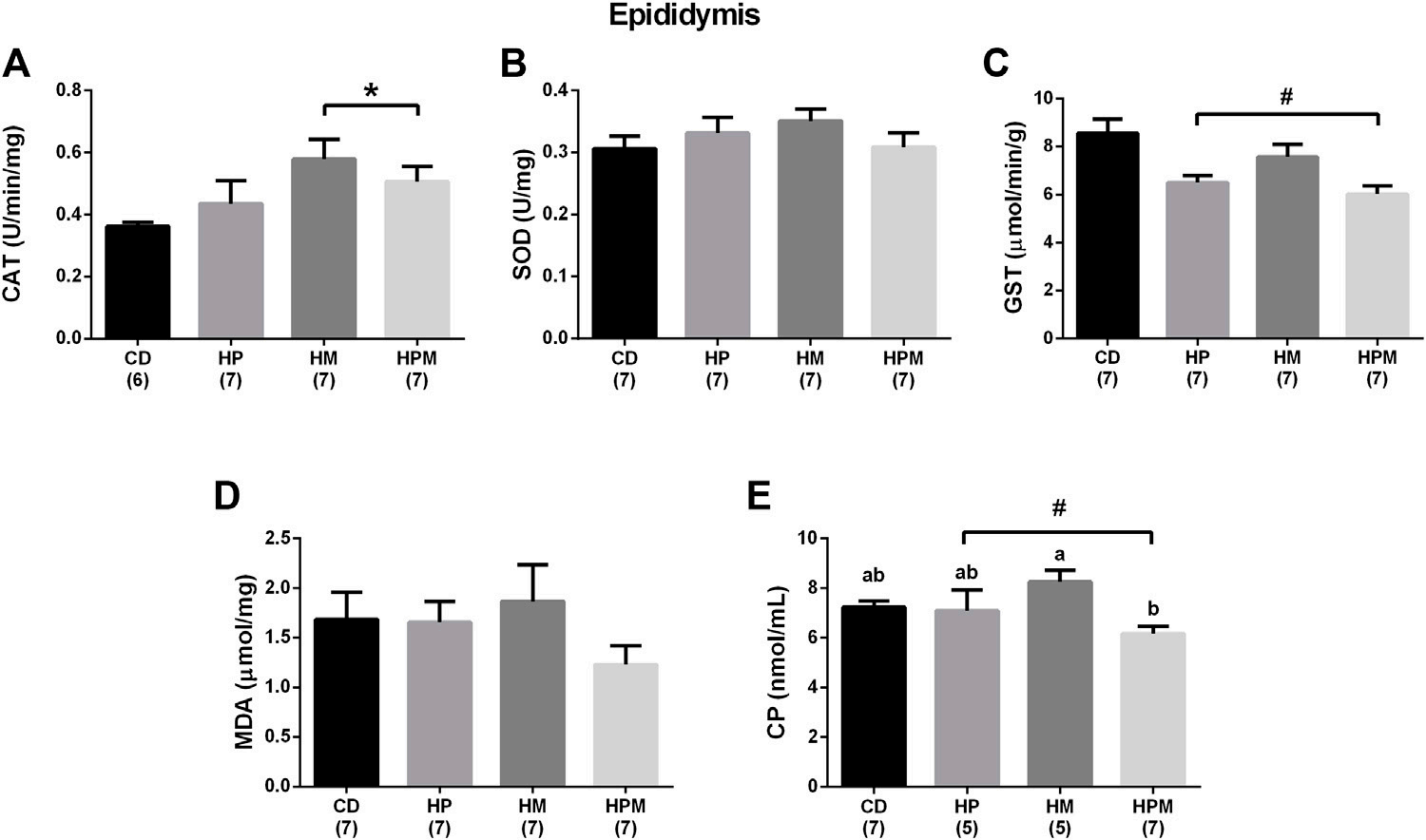
Epidydimal antioxidant enzyme activity and oxidative stress markers in male rat offspring from progenitors fed on high-fat high-sugar diet. Mean ± SEM; symbols indicate significant difference caused by parental diet (*p* < 0.05): ^#^statistically significant by the effect of paternal diet; *statistically significant by the effect of maternal diet. a,b Different letters represent significant difference between groups (*p* < 0.05). H: high-fat and high-sugar diet; CD (control paternal and maternal diet), HP (H paternal diet and control maternal diet), HM (H maternal diet and control paternal diet) and HPM (H paternal and maternal diet). **(A)**: catalase (CAT) (U/min/mg) (*n* = 6-7); **(B)**: superoxide dismutase (SOD) (U/mg) (*n* = 7); **(C)**: glutathione S-transferase (GST) (μmol/min/g) (*n* = 7); **(D)**: malondialdehyde (MDA) (μmol/mg) (*n* = 7); **(E)**: carbonyl protein (CP) (nmol/mL) (*n* = 5-7).

Our results showed significant changes in biometric data, epigenetic pattern, inflammatory response, oxidative balance, and sperm count of male offspring from female progenitors fed on high-fat high-sugar diet. There was an isolated maternal effect of diet intake on the increase in offspring body weight (*n* = 8, *p* < 0.05, Figure 1B), which could be a reflex of the increased adiposity of EPI absolute weight (*n* = 8; *p* > 0.05, Figure 2E). Adiposity could be related to the decrease in *Dnmt3a* gene expression (*n* = 4–6; *p* < 0.05, Figure 3B) and to the increase of TNF-α, IL-6, and IL-10 levels in the testis (*n* = 6–8; *p* < 0.05; Figures 4A,B,D, respectively). Along with the cytokines, the antioxidant activity of CAT and SOD was increased (*n* = 6–7; *p* < 0.05, Figures 5A,B, respectively), showing the inflammatory response that could be associated to an oxidative imbalance. As a result, sperm count and daily sperm production in the testis and sperm count in caput/corpus in epididymis was decreased by the maternal effect (Table 2). Unlike the testis, there was a decrease in TNF-α levels in the epididymis (*n* = 8; *p* < 0.05, Figure 6A), with an increase in CAT levels (*n* = 6-7; *p* < 0.05, Figure 7A).

**TABLE 2:** Sperm parameters in testis and epididymis in male rat offspring from progenitors fed on high-fat and high-sugar diet.

Sperm parameters	CD (*n* = 6)	HP (*n* = 6)	HM (*n* = 6)	HPM (*n* = 6)
Spermatid number (x 10^6^/testis)	202.4 ± 10.75^a^	156.8 ± 7.13^b^	137.6 ± 5.39^b^*	158.1 ± 6.04^b^*
Spermatid number (x 10^6^/g testis)	142.2 ± 7.70^a^	104.5 ± 6.19^ab^	90.44 ± 4.40^ab^ ^*^	101.9 ± 3.90^b^*
Daily sperm production (x 10^6^/testis/day)	33.18 ± 1.76^a^	25.70 ± 1.17^b^	22.56 ± 0.88^b^*	25.91 ± 0.99^b^*
Caput/corpus epididymis sperm number (x 10^6^/organ)	106.9 ± 8.61	114.1 ± 11.38	95.21 ± 6.90*	85.11 ± 4.4*
Caput/corpus epididymis sperm number (x 10^6^/g organ)	339.7 ± 23.64	345.8 ± 38.01	288.7 ± 17.56*	265.1 ± 15.01*
Sperm transit time in the caput/corpus epididymis (days)	3.237 ± 0.24	4.471 ± 0.47	4.215 ± 0.22	3.354 ± 0.30
Cauda epididymis sperm number (x 10^6^/organ)	134.9 ± 6.00	139.9 ± 14.52	133.9 ± 12.03	138.5 ± 8.99
Cauda epididymis sperm number (x 10^6^/g organ)	641.3 ± 30.39	635.4 ± 40.33	618.0 ± 55.06	653.8 ± 34.21
Sperm transit time in the cauda epididymis (days)	4.132 ± 0.30	5.449 ± 0.53	6.040 ± 0.73	5.431 ± 0.46
Normal shaped spermatozoa	178.0 ± 2.91	176.7 ± 2.75	180.8 ± 4.26	176.8 ± 3.73

^a,b^ Different letters represent significant difference between groups (*p* < 0.05).

Mean ± SEM., Superscript symbols indicate significant difference caused by parental diet (*p* < 0.05): *statistically significant by the effect of maternal diet. H, high-fat and high-sugar diet; CD, (control paternal and maternal diet), HP, (H paternal diet and control maternal diet), HM, (H maternal diet and control paternal diet), and HPM, (H paternal and maternal diet).

#### 3.2.2 Paternal Influence

**FIGURE 8:**
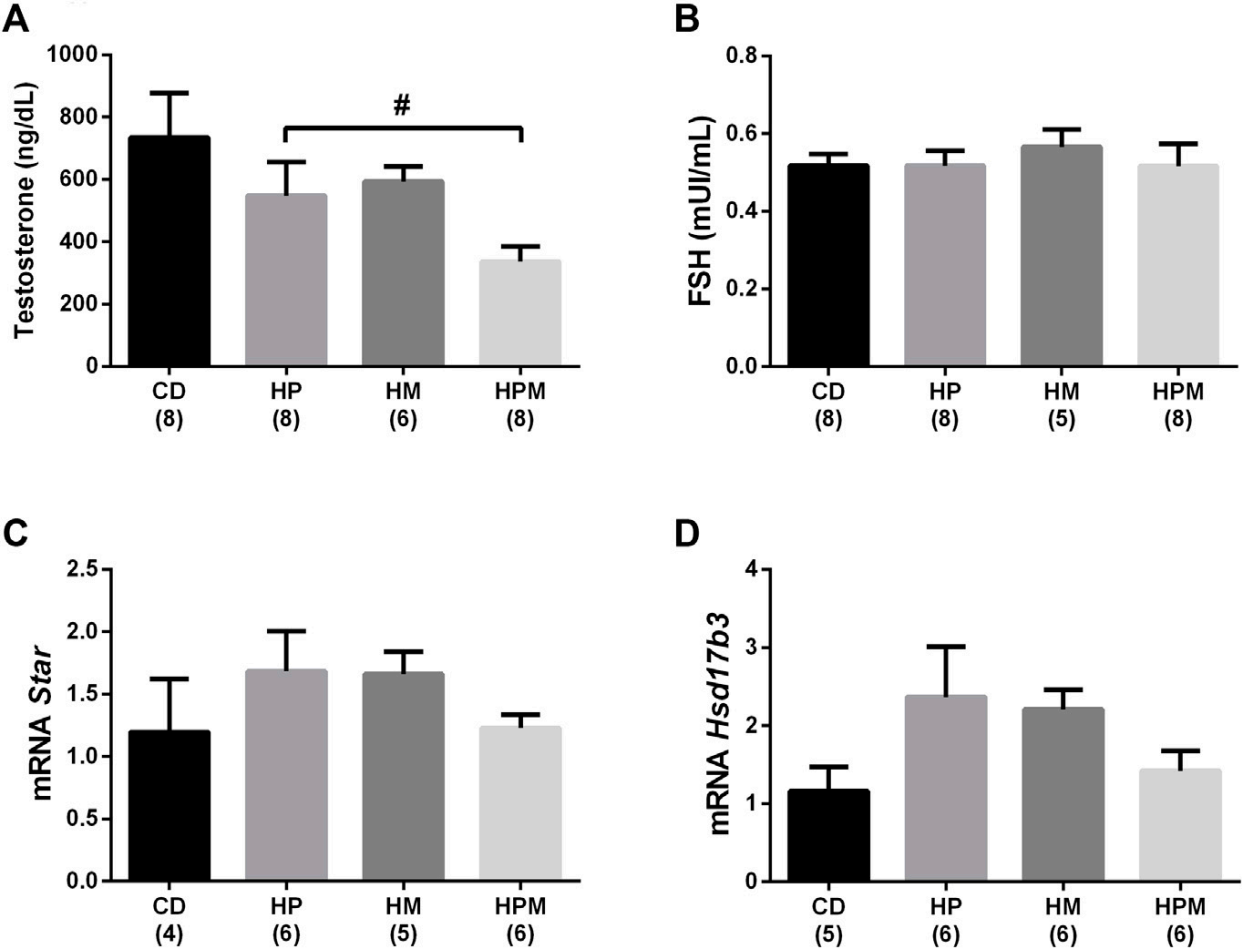
Serum levels of sex hormones and gene expression of testicular steroidogenic enzymes in male rat offspring from progenitors fed on high-fat high-sugar diet. Mean ± SEM; symbols indicate significant difference caused by parental diet (*p* < 0.05): ^#^statistically significant by the effect of paternal diet. H: high-fat and high-sugar diet; CD (control paternal and maternal diet), HP (H paternal diet and control maternal diet), HM (H maternal diet and control paternal diet) and HPM (H paternal and maternal diet). **(A)**: serum testosterone: *n* = 6–8; **(B)**: serum follicle stimulating hormone (FSH) (*n* = 5–8); **(C)**: steroidogenic acute regulatory protein (*Star*) (*n* = 4–6); **(D)**: 17β-hydroxysteroid dehydrogenase 3(*Hsd17b3*) (*n* = 5–6).

Paternal diet alone was able to influence the increase in absolute and relative weight of EPI (*n* = 8; *p* > 0.05, Figures 2E,F), along with the absolute testicular weight (*n* = 8, *p* < 0.05, Figure 1C). Increased adiposity is commonly associated to the decrease in serum testosterone levels (*n* = 6–8, *p* < 0.05, Figure 8A), as observed here in. The intake of a high-fat high sugar diet by the male progenitor decreased GST activity in the epididymis (*n* = 7; *p* < 0.05, Figure 7C), as well as the levels of the oxidative marker CP (*n* = 5–7; *p* < 0.05, Figure 7E).

#### 3.2.3 Interaction Effect of Maternal and Paternal Diet Intake

There was an interaction effect of maternal and paternal diet intake on the increase in adiposity (Figure 2). The absolute weight of RET in the HPM group was increased by the interaction effect when compared to HP and HM (*n* = 8, *p* < 0.05, Figure 2A), and the relative weight was increased in HPM when compared to HM (*n* = 8, *p* < 0.05, Figure 2B). The interaction effect also increased the absolute and relative weight of EPI in HPM group when compared to all groups (*n* = 8; *p* > 0.05, Figures 2E,F, respectively), which points towards the synergistic effect resulted from both parental intake. The interaction effect of the progenitors decreased the number of spermatids in the testis in all groups when compared to control, the number of spermatids per g testis in HPM compared to control, and also decreased the daily sperm production in all groups when compared to control (*n* = 5–6; *p* < 0.05, Table 2). Ultimately, the interaction effect reduced CP levels in HPM group when compared to HM group (*n* = 5–7; *p* < 0.05, Figure 7E).

Regarding the remaining results, there was no effect for food intake (*n* = 4), testicular relative weight, and absolute and relative epididymal weight (*n* = 8, *p* > 0.05, Figures 1A,D–F, respectively). No differences were observed for absolute and relative MES weight (*n* = 8; *p* < 0.05, Figures 2C,D) and no alterations were observed for the testicular gene expression of *Dnmt1*, *Dnmt3b*, and *Mecp2* (*n* = 5–6; *p* < 0.05, Figures 3A,C,D, respectively). In the testis, there were no changes for levels of IL-1β (*p* > 0.05, Figure 4C), GST (*n* = 7; *p* > 0.05, Figure 5C) nor for the oxidative stress markers MDA and CP (*n* = 4–7; *p* < 0.05, Figure 5D,E, respectively).

**FIGURE 9:**
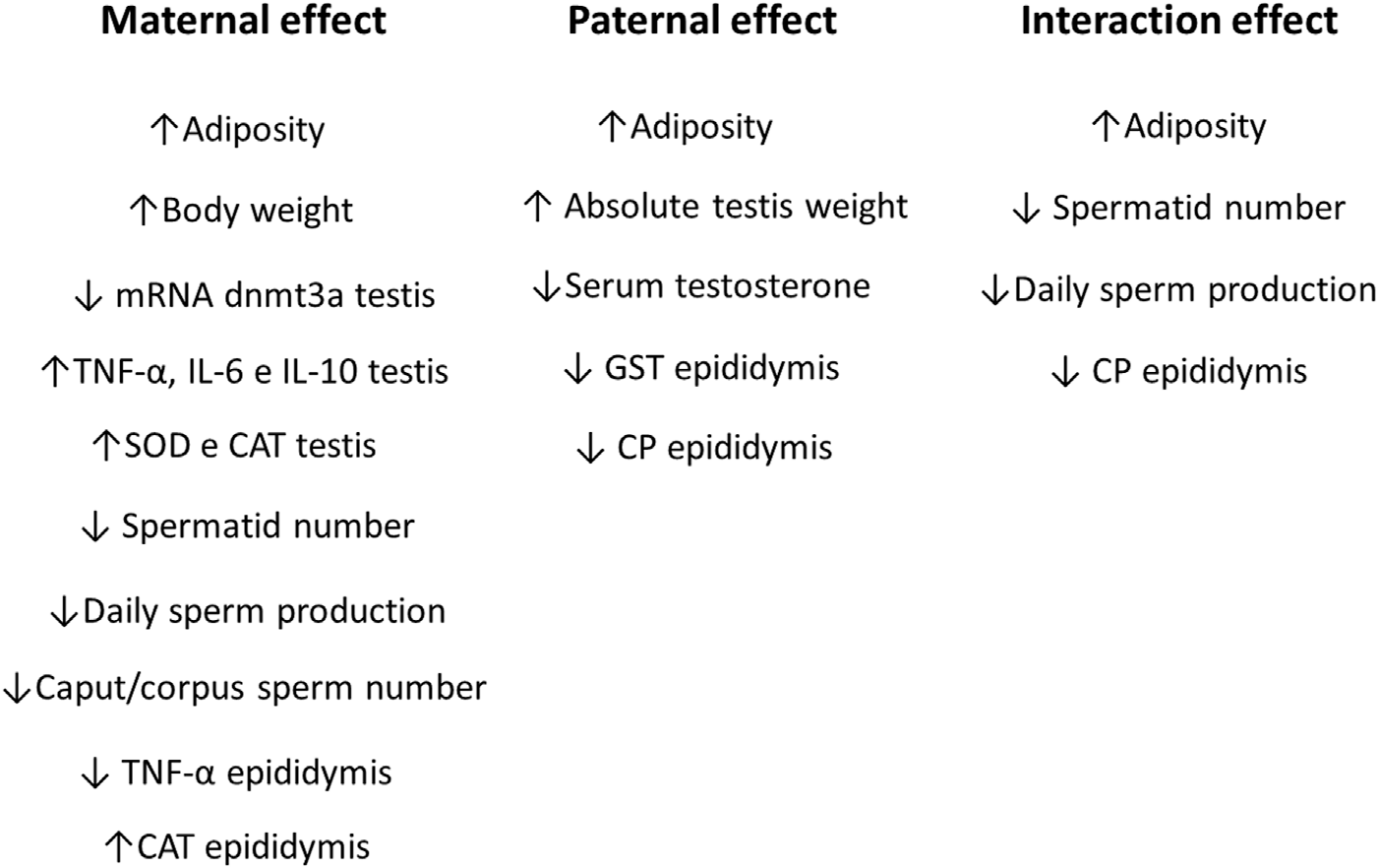
Maternal, paternal, and interaction effects in male rat offspring from progenitors fed on high-fat and high-sugar diet.

In the epididymis, no changes were observed in the levels of IL-6, IL-1β, IL-10 (*n* = 6–8; *p* > 0.05, Figures 6B–D, respectively), SOD and MDA (*n* = 7; *p* > 0.05, Figures 7B,D, respectively). There were no changes in serum FSH (*n* = 5–8; *p* > 0.05; Figure 8B) nor testicular gene expression of star and hsd17b3 (*n* = 5–6; *p* > 0.05; Figures 8C,D, respectively). The results of male offspring are summarized in Figure 9 according to the effect of each progenitor and their interaction.

## 4 Discussion

Our study evaluated the effects of parental high-fat high-sugar diet intake on the reproductive health of adult male rats. The results showed that parental diet can program epigenetic parameters, adiposity, inflammatory response, and oxidative balance in the testis of adult rats, altering daily sperm production and serum testosterone levels. To our knowledge, this is the first study to evaluate epigenetic markers and cytokine production associated to oxidative stress and sperm parameters in the testis and epididymis of male adult offspring from both parents submitted to a high-fat high-sugar diet intake.

Epigenetics comprehends heritable changes in gene expression without changes in DNA nucleotide sequence. DNA methylation is an epigenetic marker that regulates gene expression through gene silencing, a process catalyzed by DNA methyltransferases (DNMTs). DNA methylation is maintained by DNMT1 during cell replication and has been related to the maintenance of epigenetic imprinting occurred during the fetal development, while DNMT3a and DNMT3b are *de novo* DNMTs (Lyko, 2018). DNA methylation recruits methyl CpG binding proteins (MeCP), blocking RNA polymerase activity by condensing chromatin and inhibiting the binding of transcription factors, which prevents the initiation of gene expression. DNMTs induce the demethylation of the CpG sites when inactive, promoting gene expression (Allis and Jenuwein, 2016). *Dnmt3a* and *Dnmt3b* are both localized in the spermatogonial cells in rat testes and *Dnmt3a* is also expressed in spermatocytes (Xu et al., 2015). Herein, we show that maternal diet influences slightly the gene expression of the epigenetic marker *Dnmt3a* in the testis of adult offspring. DNMT3a activity can play a role in adulthood, causing modifications on pre-existing epigenetic patterns (Lyko, 2018).

Evidence suggest that high-fat diet intake could alter the DNA methylation profile in the germline of adult male rats and that could partially transmit the altered epigenetic signatures of developmental importance *via* sperm to the embryo causing embryo loss (Deshpande et al., 2020). It is known that high-fat diet intake increases adiposity in the individual per se and several studies focusing on reproductive health have shown that either maternal or paternal high-fat diet intake is able to program increased adiposity in male offspring, besides increasing adiposity in the progenitors (Palmer et al., 2012; Fullston et al., 2013; Fan et al., 2018; Deshpande et al., 2020; Sertorio et al., 2021). Our model showed that high-fat and high-sugar diet intake increased the adipose tissue weight in both progenitors and that there was an interaction effect of maternal and paternal diet intake increased adiposity of retroperitoneal and epididymal adipose tissue in adult male offspring, evidencing the synergistic effect of the paternal and maternal diets combined.

Adipocyte hypertrophy has also been associated to an activated inflammatory response in adipose tissue, producing proinflammatory cytokines such as TNF-α, IL-1 and IL-6, and C-reactive protein (Segovia et al., 2014). Some studies have shown that the exposure of the fetus to inflammatory mediators in the uterus, provoked by maternal immune stimulation, produces an offspring that exhibits a proinflammatory phenotype. This inflammatory profile is activated due to changes in the developmental programming of the immune system (Mandal et al., 2013), increasing inflammatory responses in other tissues. Consistently, we observed increased adiposity in the female progenitor in gestation, which probably set a poor environment for offspring development, programming the pattern of inflammatory response in adult male offspring. In our study, maternal diet influenced the production of the proinflammatory cytokines TNF-α and IL-6 in the testis, demonstrating that an inflammatory response has been programmed. Nevertheless, an adequate and dynamic balance between pro- and anti-inflammatory mediators must exist and evolve over time to achieve control of the cause of inflammation without inducing tissue damage (Cicchese et al., 2018). IL-10 is an anti-inflammatory cytokine that plays a counter-regulatory role in the immune response to antigens, being produced in response to proinflammatory signals to prevent excessive inflammation (Rutz and Ouyang, 2016). Accordingly, the maternal diet also altered the IL-10 levels in our study, which may reflect a testicular adaptive response in the offspring.

The inflammatory response induced by the cytokines may generate ROS and higher levels of ROS could overload the tissue’s antioxidant capacity. It could result in altered levels of the antioxidant enzymes SOD, CAT, and glutathione, and therefore, interfere with the production of testosterone, in the concentration of viable sperm and culminate in low sperm count (Azenabor et al., 2015; Tremellen, 2016; Fan et al., 2018; Yi et al., 2020). In our study, oxidative stress by maternal diet influence could be observed through the changed levels of SOD and CAT in the testis, that were altered along with the cytokines, and CAT and GST in epididymis. The oxidative imbalance did not translate into oxidative cell damage measured by this study, i.e., increased levels of MDA and CP. Another programming study reported cell damage by increased levels of MDA and nitrotyrosine in the testis and sperm when maternal exposure to a high-fat diet was prior to conception and the parameters assessed at different ages of older offspring (Rodríguez-González et al., 2015). The maternal influence and the interaction effect between both parents were able to decrease the number of spermatids in the testis, and consequently, the daily sperm production in the offspring. Other studies also showed the effects of maternal high-fat diet in male offspring related to oxidative imbalance in the testis and low daily sperm production (Sertorio et al., 2021). Impaired levels of SOD, CAT, and GPx were also observed in testis and sperm (Rodríguez-González et al., 2015; Santos et al., 2015; Bautista et al., 2017).

Besides the inflammatory response caused by higher adiposity, Sertoli cells and germ cells in the seminiferous epithelium produce cytokines, including TNF-α, TGF family members, and interleukins (Lie et al., 2013). Germ cells undergo spontaneous degeneration during spermatogenesis in order to establish the size of the cell population. This degeneration occurs through the induction of apoptosis of these cells and the breaking of the tight junctions in Sertoli cells, as well as through the inhibition of steroidogenesis in Leydig cells (Lie et al., 2013). The increased concentration of proinflamatory cytokines could intensify this degeneration process, affect the function of cell junctions that forms the blood-testicular barrier, and impair sperm production (Siu et al., 2003; Lydka et al., 2012). It is possible that the influence in concentrations of the proinflammatory cytokines TNF-α and IL-6 have also increased the degenerative process of germ cells and/or the structural function of Sertoli cells, interfering with the production of sperm numbers and, therefore, with daily sperm production. Reame et al. (2014) showed that breastfeeding is a critical period and that maternal high-fat diet intake led to cell detachment in the seminiferous tubules of the offspring, leading to impairment in daily sperm production.

The increased adiposity caused by parental high-fat diet intake could still impact the hypothalamic-pituitary-gonadal axis of the male progeny, leading to hormonal disruption and predisposing the offspring to the development of central hypogonadism (Crean and Senior, 2019). High-fat intake results in the deregulation of adipokines, such as leptin, that plays an important role in reproductive health by stimulating gonadotropin-releasing hormone (GnRH) release (Tsatsanis et al., 2015). Testosterone is the main male steroid hormone and its production is dependent on the production of GnRH by the hypothalamus. GnRH activates in the pituitary gland the production of prolactin, LH and FSH, the hormones that regulate reproductive function.

LH activates the expression of StAR, that is responsible for cholesterol transport into the mitochondria in Leydig cells; the cholesterol side-chain cleavage enzyme (P450scc) then produces pregnenolone, which is converted into progesterone by enzymes of the smooth endoplasmic reticulum. Progesterone is converted into testosterone by 17β-HSD (Ross and Pawlina, 2011; Kierszenbaum and Tres, 2016). The expression of *Star* and *Hsd17b3* were not significantly altered in our study, and that could be due to the age of the animals or the type of diet that the offspring consumed during life. A study showed that male offspring 120 days-old from obese fathers and submitted to high-fat diet intake over life showed decreased gene expression of *StAR*, *P450scc*, and *17β-HSD* and consequently low circulating levels of testosterone when compared to male offspring from lean fathers (Sanchez-Garrido et al., 2018). Changes in serum testosterone levels due to paternal diet influence were observed in our study, which corroborates with several reproductive programming studies reporting the decrease of circulating levels of testosterone in male offspring of mothers or fathers fed an high-fat diet (Jacobs et al., 2014; Reame et al., 2014; Rodríguez-González et al., 2015; Navya and Yajurvedi, 2017; Mao et al., 2018; Sanchez-Garrido et al., 2018).

Another common feature observed in studies on reproductive programming is the lower sperm count in the cauda epididymis and vas deferens due to maternal or paternal high-fat diet intake (McPherson et al., 2014; Reame et al., 2014; Fullston et al., 2015; Rodríguez-González et al., 2015; Santos et al., 2015; Navya and Yajurvedi, 2017; Mao et al., 2018; Youngson et al., 2019). The spermatids produced by the testis pass through the epididymis, where the sperm acquisition of motility, the ability of sperm to fertilize, and the storage of sperm until ejaculation occurs. The exposure of these cells to a healthy epididymal microenvironment is crucial for maturation, which is also regulated by the transit time of sperm through the organ (Sertorio et al., 2021). Our results showed there were changed levels of CAT and in the production of the proinflammatory cytokine TNF-α in cauda epididymis due to maternal high-fat high-sugar intake. An interaction effect was observed in lower CP levels, an oxidative protein marker. Although maternal diet had changed the sperm number in caput/corpus epididymis, there were no changes in sperm transit time in this region. The changed sperm count in caput/corpus epididymis would probably be due to the low spermatid count in the testis. The changed CAT levels were probably able to fight the activation of the inflammatory response in cauda epididymis, influencing TNF-α and CP levels. Unbalanced levels of GST were observed due to paternal diet, and altogether, could have reflected the lack of change in sperm count and transit time in cauda epididymis, as well as maintaining the proportion of morphologically normal sperm from the vas deferens. In male offspring exposed to the maternal obesogenic environment at different stages of development, the epididymis transit time was also not altered (Reame et al., 2014).

Reproductive programming studies showed lower activity of the antioxidant enzymes, increased levels of stress markers, decreased number of normal sperm and sperm motility in the sperm of offspring of mothers submitted to high-fat diet intake (Rodríguez-González et al., 2015; Santos et al., 2015). Paternal high-fat diet intake was also shown to damage sperm parameters in offspring (Fullston et al., 2012; McPherson et al., 2014; Fullston et al., 2015). Although sperm count, transit time, and sperm morphology remained unchanged in our study, at a molecular level the cytokine production, activity of antioxidant enzymes and oxidative stress marker point out the interaction effect of maternal and paternal diet. According to our results, it is possible to suggest that the maternal effect is more pronounced than the paternal effect. That could be explained by the differences in paternal and maternal epigenetic inheritance, since maternal epigenetic reprogramming can occur at different temporal windows and alter more directly the offspring environment—the periconceptional environment, affecting early embryo reprogramming; the erasure and reacquisition of primordial germ cells during gestation; and the postnatal period (Radford, 2018). On the other hand, paternal epigenetic inheritance can be transmitted only through sperm, what could reflect the less pronounced effect observed in the offspring phenotype.

## 5 Conclusion

Parental high-fat high-sugar diet intake prior to conception and/or during gestation and lactation could influence the reproductive health of male offspring that consumed control diet after weaning at a molecular level, showing the potential programming effects of isolated or combined intake, and that the maternal effect seems to be more pronounced than the paternal effect. High-fat high-sugar diet when consumed by the mother during gestation and lactation could affect the testicular epigenetic parameter, inflammatory response, oxidative balance, and daily sperm production of the offspring in early adulthood. The lower daily sperm production effect could be exacerbated by the interaction with the high-fat high-sugar diet consumed by the father prior to conception. Paternal diet influenced lower serum testosterone levels, which could have played a role in daily sperm production of the offspring. High-fat high-sugar diet intake by the parents have also altered the inflammatory response and antioxidant enzyme activity in the cauda epididymis. The alteration in the production of pro-inflammatory cytokine in the epididymis and the oxidative stress marker probably prevented deleterious effects in sperm at a morphologic level.

## Data Availability

The raw data supporting the conclusions of this article will be made available by the authors, without undue reservation.
