# Supplementary figures and images for: Parental High-Fat High-Sugar Diet Intake Programming Inflammatory and Oxidative Parameters of Reproductive Health in Male Offspring
Source: Front Cell Dev Biol. 2022 Jun 27;10:867127. doi: 10.3389/fcell.2022.867127 (PMC9271829; doi:10.3389/fcell.2022.867127)

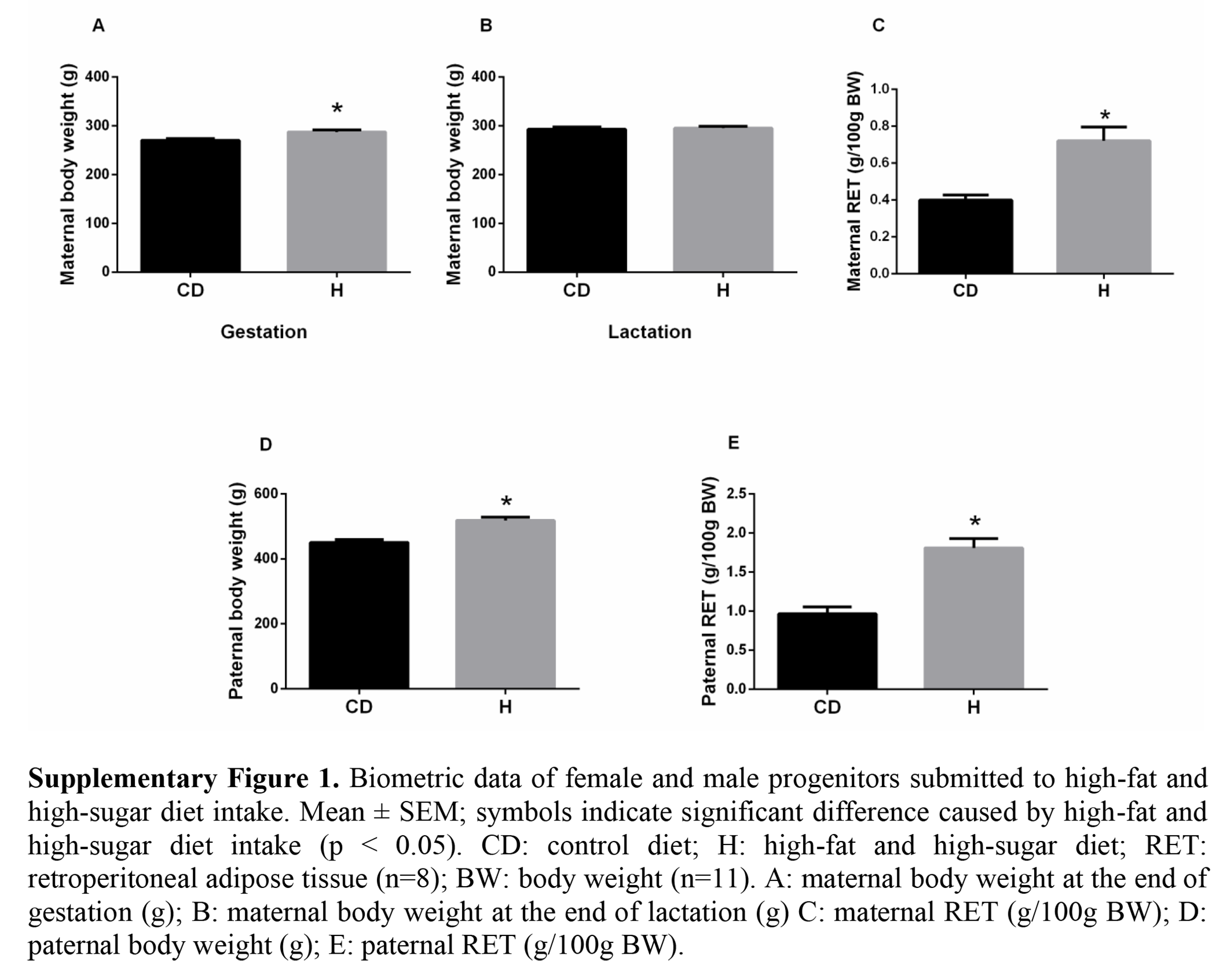

Supplement: Supplementary file 1 [file Image1.tif]
